# An economic evaluation of intensive hypertension control in CKD patients: a cost-effectiveness study

**DOI:** 10.1186/s40885-022-00215-4

**Published:** 2022-11-01

**Authors:** Ye Seol Lee, Hae-Young Lee, Tae Hyun Kim

**Affiliations:** 1 Department of Rehabilitation Medicine, Seoul National University Hospital, Seoul National University College of Medicine, Seoul, Republic of Korea; 2 National Traffic Injury Rehabilitation Research Institute, National Traffic Injury Rehabilitation Hospital, Yangpyeong, Republic of Korea; 3 Division of Cardiology, Department of Internal Medicine, Seoul National University Hospital, Seoul National University College of Medicine, Seoul, Republic of Korea; 4 Department of Healthcare Management, Graduate School of Public Health, Yonsei University, Seoul, Republic of Korea

**Keywords:** Economic evaluation, Intensive hypertension control, Cost-effectiveness, Chronic kidney disease

## Abstract

**Background:**

Studies have suggested that intensive hypertension control in patients with a high risk of cardiovascular disease (CVD) is both effective and economically feasible. The purpose of this study is to conduct an economic evaluation of intensive hypertension control targeting chronic kidney disease (CKD) patients using the representative data in Korea.

**Methods:**

We used a Markov decision model to compare both cost and effectiveness of intensive hypertension control versus standard hypertension control in hypertensive CKD patients. Model parameters were estimated with the data from the National Health Insurance Service (NHIS)-National Sample Cohort, as well as latest literature. One-way sensitivity analysis was conducted to test the effect of variation in key parameters on the model outcome.

**Results:**

For CKD patients with hypertension, intensive hypertension control would cost more but increase utilities, compared to standard hypertension control. The incremental cost-effectiveness ratio (ICER) for intensive hypertension control in CKD patients was projected at 18,126 USDs per quality-adjusted life year (QALY) compared to standard hypertension control. The results of sensitivity analysis suggest that the results are overall robust.

**Conclusions:**

This study finds that intensive hypertension control in CKD patients in Korea is economically sound. This information is expected to be useful for clinicians in managing hypertension of CKD patients and policymakers when making decisions.

## Background

Adults with hypertension are on the rise in Korea. Because of the high prevalence of hypertension among adults, Korea faces a huge population health concern. It is estimated that there are more than 12 million hypertensive patients in Korea, of which only less than 50% are controlling their blood pressure (BP) [1].

Hypertension is a well-known risk factor for cardiovascular disease (CVD) and the progression of chronic kidney disease (CKD) in people with CKD [2–4]. Therefore, control of hypertension is particularly important for the patients.

Recent studies suggest that intensive BP lowering benefits patients. The Systolic Blood Pressure Intervention Trial (SPRINT) discovered that for individuals with high CVD risk, lowering systolic BP to 120 mmHg rather than the normal 140 mmHg reduced CVD events by 25% and all-cause death by 27% [5]. A review article also found that intensive hypertension control helps patients lower their BP and decrease the risk of major CVD events [6].

While previous studies targeted various hypertensive patients, a paper published by the SPRINT research group suggests that it intensive BP management is particularly effective in hypertensive CKD patients [7].

However, when adopting intensive hypertension control, additional costs would incur to patients and society. Adverse events also might occur. However, the literature suggests that intensive hypertension control is cost-effective for most target populations in the United States as well as China, despite the fact that it usually costs more and increases the chance of adverse events, compared to standard control [8–10].

The purpose of this study is, therefore, to evaluate whether it is economically sound if the same strategy is applied to CKD patients in Korea, and to provide both clinicians and policymakers information regarding hypertension treatment strategies.

## Methods

### Data

The data were obtained from the National Health Insurance Service (NHIS) with a retrospective cohort representing Korean adults 20-year-old and over that was stratified by gender, age, subscriber classification, insurance premium quintile, and region from 2016 to 2020, and sampled by 10%. We used the same extraction method as the general sample cohort provided by the NHIS.

### Research subjects

We set the study subjects as hypertensive CKD patients aged 50 or older with systolic BP between 130–180 in 2018, which is same as the condition of including SPRINT study subjects. In addition, we incorporated the examination data to confirm whether the risk of CVD is high according to SPRINT standards (age, systolic BP, eGFR, Framingham Risk Score). We calculated CVD incidence, death, and medical expenses for 2019–2020.

### Economic evaluation model

**Fig. 1:**
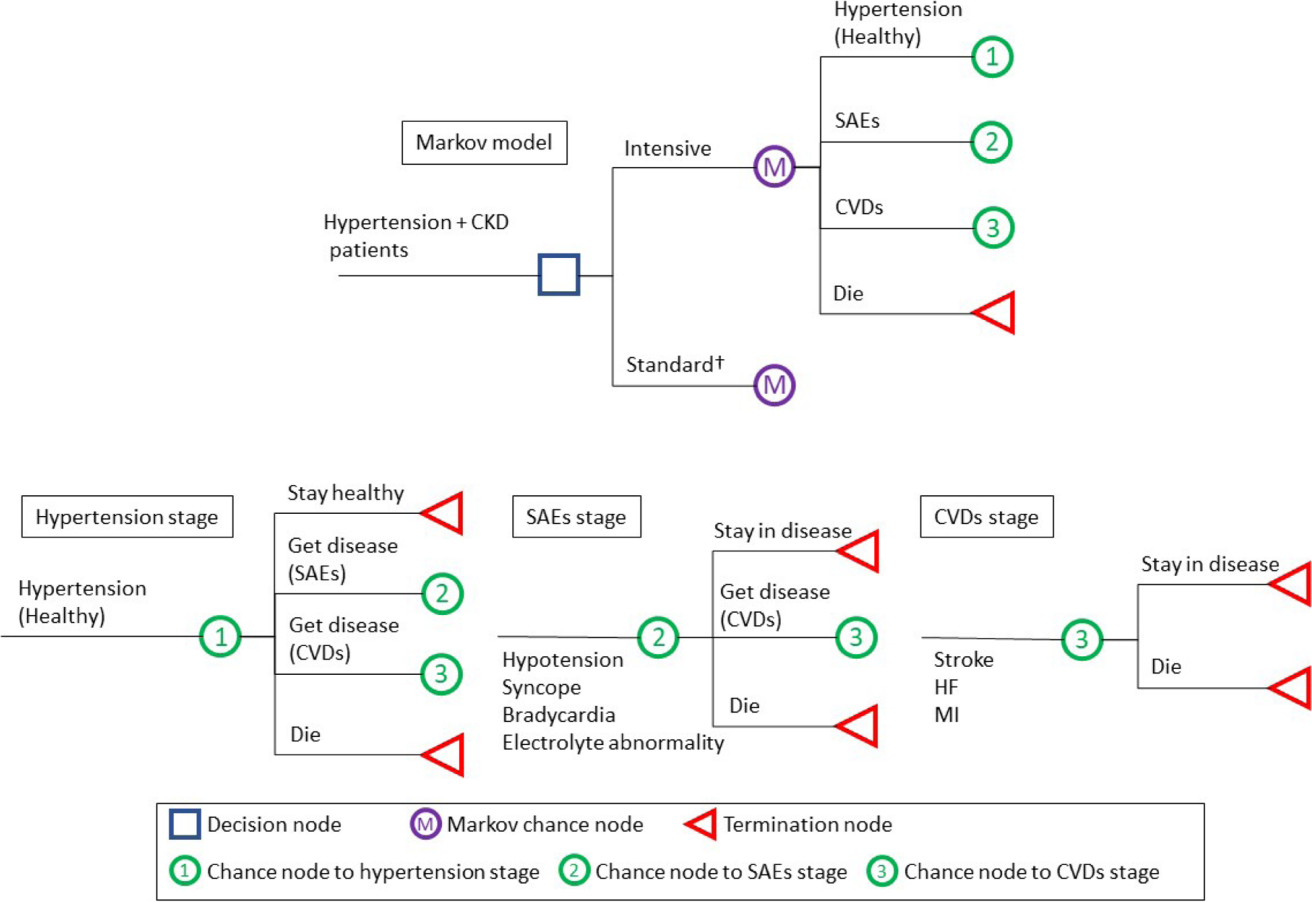
Markov decision model depicting hypertension control for CKD CKD, chronic kidney disease; SAE, serious adverse event; CVD, cardiovascular disease; HF, heart failure; MI, myocardial infarction. † The Markov node of "Standard" treatment is the same on that of "Intensive" treatment

We developed a Markov decision model to conduct a cost-effectiveness analysis (CEA) of intensive hypertension control for the CKD patients with hypertension aged 50 or older. This age group was chosen based on evidence from previous clinical trials [5]. The model's conceptual framework is depicted in Fig. 1. It is assumed that a patient with hypertensive CKD can be placed on either of the two strategies: intensive vs. standard hypertension control.

Then, the patient can progress to one of four states: hypertensive CKD (healthy), serious adverse events (SAEs), CVDs, and death. Following the literature, SAEs due to side effects from anti-hypertensive drugs include hypotension, syncope, bradycardia, electrolyte abnormalities, injurious fall, and acute renal failure (The SPRINT Research Group, 2015). CVDs include myocardial infarction (MI), stroke, and heart failure (HF). If a CVD or other unfavorable event is critical, then one may die right away, or he/she may survive and continue to remain in the CVD state. In the model, each health conditions have yearly costs and quality-of-life utilities that increase over time. A transition probability, which changes depending on demographic variables and the hypertension control approach used, defines movement between any two health states. The basic simulation cycle was 1 year. TreeAge Pro software was used to build the model (TreeAge Software, Inc., Williamstown, MA, USA).

### Model parameters

To obtain the parameters for the Markov decision model, we estimated the rates of incidence and mortality of CVDs and SAEs, and their costs using the NHIS database. In addition, we reviewed recent publications [5–7] to derive hazard ratios for intensive vs. standard hypertension control, and utilities of each health states [11] measured by quality-adjusted life years (QALYs). These parameters and sources are shown in Table 1.

**Table 1:** Input parameters and sources for the cost-effectiveness analysis model

Parameter	Data	Source
Incidence, among hypertension patients (age dependent)		
Myocardial infarction	0%–0.82%	NHIS database 2019–2020
Stroke	2.47%–6.04%	
Heart failure	2.47%–12.75%	
Hypotension	0%–0.83%	
Syncope	0%–2.01%	
Bradycardia	0%–1.32%	
Electrolyte abnormality	2.01%–3.31%	
Injurious fall	34.23%–37.86%	
Acute renal failure	0.41%–4.70%	
Mortality, among hypertension patients (age dependent)		
Hypertension	0.04%–0.53%	NHIS database 2019–2020
Myocardial infarction	0%	
Stroke	0%–0.67%	
Heart failure	0%–2.01%	
Hypotension	0%–0.41%	
Syncope	0%–0.28%	
Bradycardia	0%	
Electrolyte abnormality	0%–0.26%	
Injurious fall	0.41%–1.34%	
Acute renal failure	0%–2.01%	
Hazard ratio (Intensive vs. Standard treatment)	HR (95% CI)	
Disease events	0.94 (0.62–1.44)	
Myocardial infarction	1.35 (0.60–3.08)	Cheung, et al [7] 2017
Stroke	0.99 (0.57–1.70)	
Heart failure	0.72 (0.47–1.10)	
Hypotension	1.34 (0.88–2.04)	
Syncope	1.28 (0.86–1.92)	
Bradycardia	0.92 (0.59–1.44)	
Electrolyte abnormality	1.35 (0.94–1.94)	
Injurious fall	0.90 (0.71–1.15)	
Acute renal failure	1.46 (1.10–1.95)	
Cardiovascular disease death	0.57 (0.31–1.02)	
Non-cardiovascular disease death	0.72 (0.53–0.99)	
Costs (per person, per 1-year, US$, age dependent)		
Hypertension	386.4–1,943.3	NHIS database 2019–2020
Myocardial infarction	159.8–26,696.9	
Stroke	2,406.1–14,109.8	
Heart failure	4,508.8–6,371.5	
Hypotension	67.0–7,930.4	
Syncope	494.6–2,882.2	
Bradycardia	365.0–3,643.9	
Electrolyte abnormality	643.6–5,511.5	
Injurious fall	4,157.2–14,087.9	
Acute renal failure	5,418.7–27,676.9	
Utility (quality-adjusted life year)		
Hypertension	1.00	Sullivan et al [11] 2006
Stroke	0.65	
Myocardial infarction	0.70	
Heart failure	0.64	
Serious adverse events	0.60	

*NHIS* National Health Insurance Service, *HR* hazard ratio, *CI* confidence interval

### Cost estimation

From the insurer's point of view, we included direct medical costs only, and thus, did not include direct non-medical costs (e.g., transportation costs) and indirect costs (e.g., time lost from work). Direct medical costs consist of hospitalization costs and outpatient costs (including prescriptions). We assumed that intensive hypertension control would incur more outpatient costs (about 1.2 times) than standard hypertension control. We derived those estimates from the National Health Insurance database. All costs were converted into US dollars (USDs), an exchange rate of 1 USD to 1,180 Korean won was applied.

### Alternatives

We compared standard hypertension control, which involves lowering systolic/diastolic BP to 140/90 mmHg; and intensive hypertension control, which entails reducing systolic/diastolic BP to 120/80 mmHg based on the SPRINT [5].

### Markov chain simulation

A cohort of 72,429 hypertensive CKD patients was simulated. It was assumed that they would take either intensive or standard hypertension control and would progress through SAEs, CVD events, and death over the lifetime. To compute present values, both costs and effectiveness (QALYs) were discounted at a 5% annual rate. One-way sensitivity analysis and tornado analysis were used to assess the robustness of our findings to various variables such as discount rates and relative risks (RRs).

## Results

**Fig. 2:**
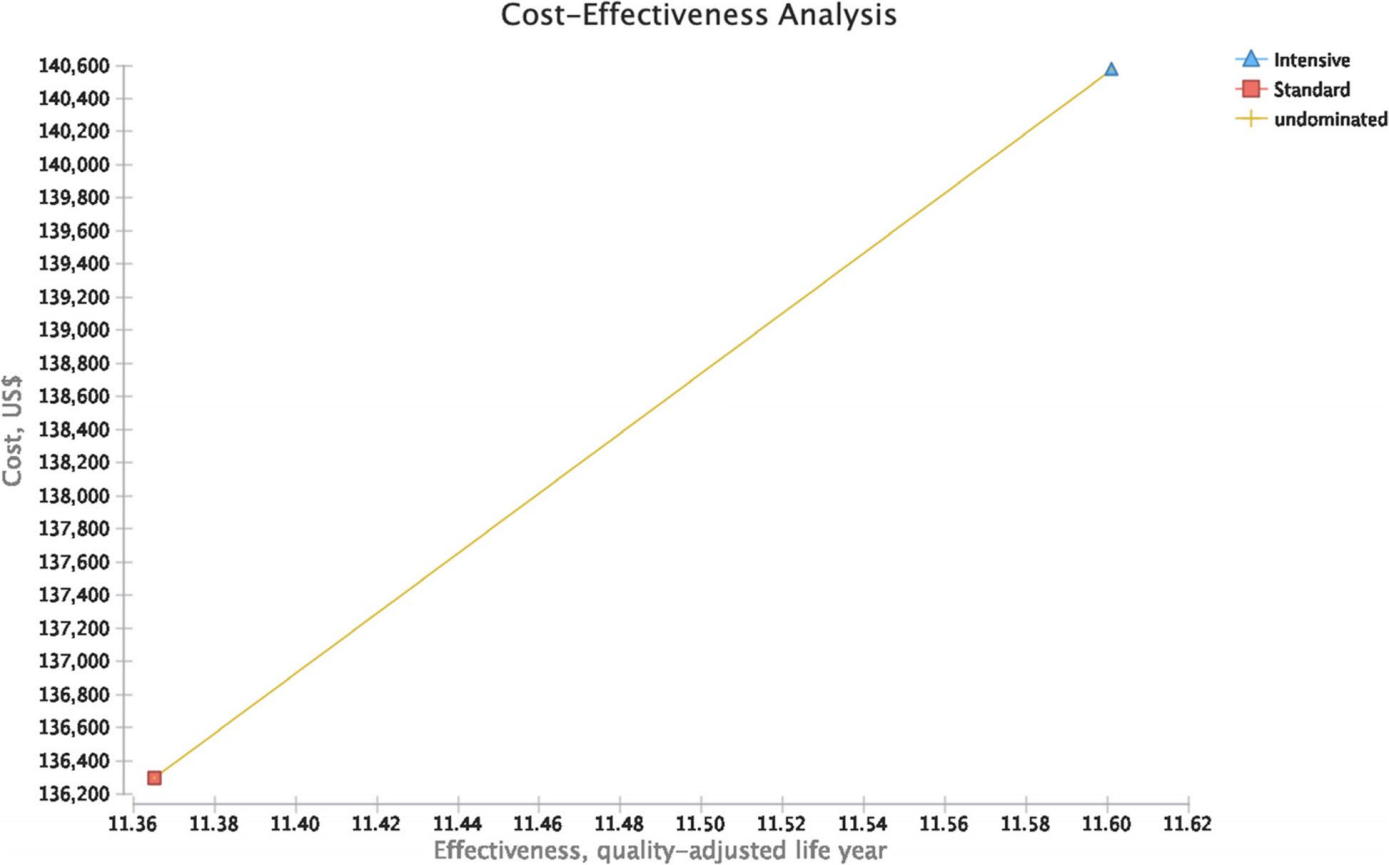
Cost-effectiveness plot for intensive vs. standard hypertension control

Table 2 presents the results for cost-effectiveness for both intensive and standard hypertension control strategies. Standard control costs 136,298.1 USDs, and gains 11.37 QALYs, and thus, its cost per effectiveness is 11,992.6 USDs. On the other hand, intensive control costs 140,572.4 USDs, and gains 11.60 QALYs. Figure 2 shows a graph for the alternatives. Since both intensive and standard hypertension control strategies are undominated, they both can be considered from an economic perspective.

**Table 2:** Cost-effectiveness results

Intervention	Cost (US$)	Incremental Cost (US$)	Effectiveness (QALY)	Incremental Effectiveness (QALY)	Incremental cost effectiveness ratio (US$)	Cost/effectiveness (US$)	Category of dominance
Standard	136,298.1	-	11.37	-	-	11,992.6	undominated
Intensive	140,572.4	4,274.3	11.60	0.24	18,125.7	12,117.2	undominated

*QALY* quality-adjusted life year

However, because intensive control is both more expensive and more effective, we calculated both incremental cost and incremental effectiveness, which are 4,274.3 USDs and 0.24 QALYs, respectively (Table 2). Therefore, the incremental cost-effectiveness ratio (ICER) for intensive hypertension control in CKD patients was projected at 18,125.7 USDs per QALY compared to standard hypertension control.

The results of one-way sensitivity analysis are shown in Table 3. When changing the discount rates to 3% and 7% rather than 5%, the ICERs for intensive hypertension control were 18,497.1 USDs and 17,269.1 USDs, respectively. This suggests that the results are overall robust regardless of different discount rates.

**Table 3:** One-way sensitivity analysis

Discount rate	Intervention	Cost (US$)	Incremental Cost (US$)	Effectiveness (QALY)	Incremental Effectiveness (QALY)	Incremental cost effectiveness ratio (US$)	Cost/effectiveness (US$)
3%	Standard	195,938.1	-	15.39	-	-	12,732.0
	Intensive	203,133.1	7,195.0	15.78	0.39	18,497.1	12,117.2
7%	Standard	101,291.2	-	8.90	-	-	11,374.8
	Intensive	104,023.4	2,732.1	9.06	0.16	17,269.1	11,477.7

*QALY* quality-adjusted life year

**Fig. 3:**
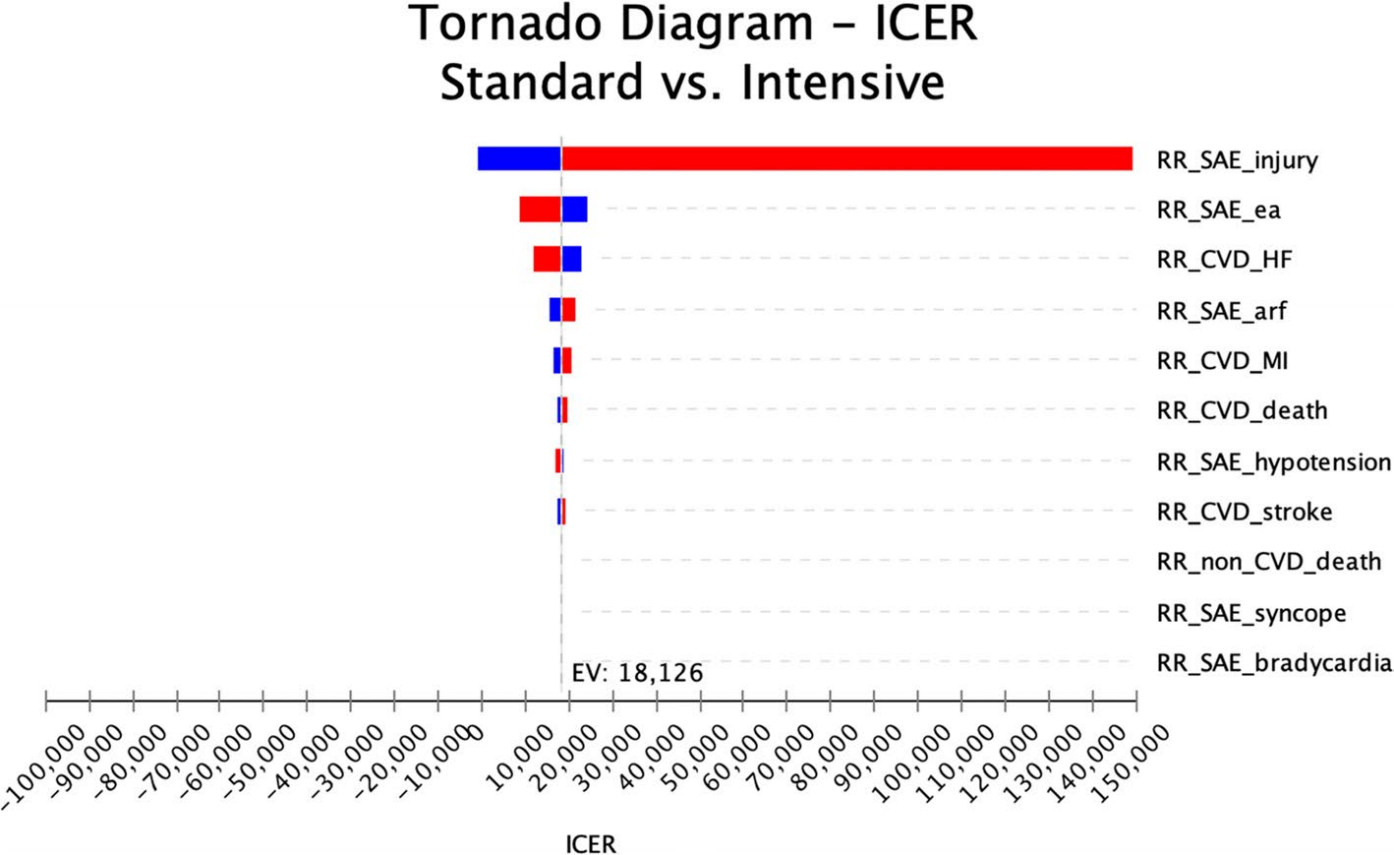
Tornado diagram. ICER, incremental cost-effectiveness ratio; RR, relative risk; SAE, serious adverse event; CVD, cardiovascular disease; HF, heart failure; MI, myocardial infarction

In addition, we produced a Tornado diagram (Fig. 3) which shows sensitivity of results depending upon the different parameter values for RR for SAEs, CVDs, and death. The tornado diagram displays the relative importance of parameters. Although each variable is uncertain to some extent, the top one bar, RR for injurious fall in this study, represents that it had the greatest impact on ICERs, while others have modest effect.

## Discussion

We conducted a cost-effectiveness analysis of intensive hypertension control for hypertensive CKD patients. Since high-risk patients benefit the most from intensive hypertension control [6], we focused on CKD patients with hypertension in this study.

We discovered that, compared to standard hypertension control, intensive hypertension control could prevent more CVD events for hypertensive CKD patients in Korea. We employed a Markov model of hypertension disease progression and adjusting the model using the representative data of Korean patients. Following a recent study [12], we used the gross domestic product (GDP) per capita in Korea in 2019 (approximately $31,362.80 per QALY) as the willingness-to-pay threshold for the ICER. Since intensive hypertension control did not exceed such threshold, one might determine that it is worthwhile to spend, and thus, economically reasonable. The result of sensitivity also analyses suggest that the use of intensive hypertension control for CKD patients is still cost-effective after considering different discount rates or key parameters except the case of serious event, such as injurious fall.

Since we followed recent studies in which the effect of intensive hypertension control was examined [7, 8], our findings may be generalizable and reflect the most recent evidence. We had access to more recent data and were able to conduct more detailed analyses for various subgroups. In addition, the result of this study is in line with recent publications that report cost-effectiveness of intensive BP control [9, 10, 13].

However, like other studies, ours has limitations in the simulation model. For example, while we projected incidences of SAEs, CVDs, and death based on the Korean population, we applied relative ratios for such outcomes based on the published article in foreign country, such as US in the model due to a lack of published data in Korea for risk estimation.

Because the data are sample, some serious adverse effects (e.g., syncope, bradycardia, and acute kidney injury) may have been under-represented. Furthermore, we only compared two strategies: intensive vs. standard. But, there may be other strategies in managing hypertension among CKD patients.

Utilities of health states may have impact on overall outcomes and ICERs. But, due to limited data, we could not conduct sensitivity analyses based on varying values for QALYs for each health state.

We could not incorporate health behavioral factors such as diet and smoking either. Our estimations may have underestimated the burden of CVD among CKD patients. Also, future studies would benefit if they assess the cost-effectiveness of intensive hypertension control with varying patient adherence rates.

Intensive hypertension control would result in an increase in direct medical costs for both patients and payers perspective, savings through lowering significant adverse events and enhanced quality of life can justify such strategy.

As the literature suggests that intensive hypertension control of CKD patients is more beneficial for older persons and for males, due to an increasing risk of CVD among older persons [10], we would think that it may also be the case for this study’s subjects.

## Conclusions

In conclusion, this study finds that intensive hypertension control in CKD patients is economically reasonable. Our findings should be useful for both clinicians and policymakers when setting priorities for intensive hypertension control.

## Data Availability

Not applicable.

## Abbreviations

BP: Blood Pressure
CVD: Cardiovascular Disease
CKD: Chronic kidney Disease
NHIS: National Health Insurance Service
CEA: Cost-effectiveness Analysis
SAE: Serious Adverse Event
QALY: Quality-Adjusted Life Years
RR: Relative Risk
ICER: Incremental Cost-Effectiveness Ratio
